# Clinical Characteristics, Pathogen Distribution, and Factors Affecting Visual Outcomes of Pediatric Post-Traumatic Endophthalmitis

**DOI:** 10.3390/antibiotics14010020

**Published:** 2025-01-02

**Authors:** Xiaoxia Li, Yibin Zhou, Zhi Chen, Xiuwen Zhang, Zimei Zhou, Maureen Boost, Taomin Huang, Xingtao Zhou

**Affiliations:** 1 Department of Pharmacy, Eye & ENT Hospital, Fudan University, Shanghai 200031, China; xiaoxia.li@fdeent.org (X.L.); 22211260025@m.fudan.edu.cn (Y.Z.); xiuwen.zhang@fdeent.org (X.Z.); 2 Eye Institute and Department of Ophthalmology, Eye & ENT Hospital, Fudan University, Shanghai 200031, China; peter459@aliyun.com; 3 NHC Key Laboratory of Myopia (Fudan University)—Key Laboratory of Myopia, Chinese Academy of Medical Sciences, Shanghai 200031, China; 4 Shanghai Key Laboratory of Visual Impairment and Restoration, Shanghai 200031, China; 5 Department of Ophthalmology, BronxCare Health System, Bronx, NY 10456, USA; zzhou@bronxcare.org; 6 School of Optometry, Hong Kong Polytechnic University, Hong Kong SAR 999077, China

**Keywords:** pediatric post-traumatic endophthalmitis, pars plana vitrectomy (PPV), best-corrected visual acuity (BCVA), retinal detachment, norvancomycin

## Abstract

**Objective:** This study aimed to investigate the etiology, pathogens, antibiotic susceptibility, treatments, and factors influencing the visual prognosis of pediatric post-traumatic endophthalmitis (PTE) to provide valuable insights for clinical diagnosis and treatment. **Results:** A total of 301 children were included, with 142 (47.2%) cultures yielding positive results. Gram-positive cocci were the predominant pathogens (71.1%), with high sensitivity to vancomycin (95.4%). Pars plana vitrectomy (PPV) was performed in 216 eyes (71.8%), with emergency or immediate vitrectomy within 24 h of hospitalization performed on 171 eyes (56.8%). The first intravitreal antibiotic injection, consisting of ceftazidime and norvancomycin, was administered to 248 patients (82.4%). The absence of retinal detachment (OR, 0.191; 95% CI, 0.065–0.560; *p* = 0.002), normal intraocular pressure (OR, 1.894; 95% CI, 1.151–3.117; *p* = 0.012), and no lens extraction (OR, 0.187; 95% CI, 0.069–0.504; *p* < 0.001) were found to be independent factors associated with better visual outcomes (BCVA) in pediatric PTE patients. **Methods:** A retrospective analysis was conducted on pediatric PTE patients treated between January 2012 and June 2022. Data were collected on clinical characteristics, causative pathogens, antibiotic sensitivity, treatments, and visual outcomes. **Conclusions:** Gram-positive cocci are the most common pathogens in pediatric PTE, with early vitrectomy and intravitreal ceftazidime and norvancomycin being the most effective treatments. Favorable visual outcomes are strongly associated with the absence of retinal detachment, normal intraocular pressure, and no lens extraction. These findings highlight the need for timely surgical and antimicrobial interventions tailored to each patient to improve visual prognosis.

## 1. Introduction

Open globe injury (OGI) is a significant cause of visual morbidity and monocular vision loss across all age groups. While endophthalmitis is a rare complication of OGI, it poses a severe threat to vision. In cases where OGI is complicated by endophthalmitis, the management and visual outcomes are often more complex compared to trauma alone [1,2,3]. The incidence of pediatric post-traumatic endophthalmitis (PTE) following OGI varies considerably across different regions, with reported rates ranging from 4.9% to 54.2% [4,5]. Notably, PTE occurs at a rate ten times higher than that of postoperative endophthalmitis, with risk further elevated by the presence of an intraocular foreign body (IOFB), ranging from 6% to 30% [1,6,7]. Additionally, injuries involving Zone I (the cornea and limbus) are particularly associated with a higher risk of infection, as wounds in this region provide a direct pathway for microbial entry into the eye, thereby increasing susceptibility to endophthalmitis [4,8].

The clinical presentation of post-traumatic endophthalmitis is highly variable, making it challenging to distinguish it from traumatic inflammatory responses [9,10]. Timely clinical examination and the early initiation of prophylactic antibiotics are critical, as delayed intervention worsens outcomes [11,12,13]. Key clinical signs, such as increasing pain, corneal infiltration, retinitis, and vitritis, should raise suspicion of endophthalmitis [14]. Bacillus endophthalmitis, in particular, is known for its rapid progression, while fungal endophthalmitis tends to follow a more subacute course [15,16].

Bacterial infections are the primary cause of post-traumatic endophthalmitis, although fungal pathogens can occasionally be involved [17]. Culture-positive and culture-negative post-traumatic endophthalmitis ranges from 0.8% to 10% and 1.6% to 12.9%, respectively [6]. Staphylococcus, Streptococcus, and both Gram-positive and Gram-negative bacilli are commonly implicated. Soil-contaminated IOFBs often involve Gram-positive bacilli, while coagulase-negative Staphylococcus (*S. epidermidis*) is more frequently found in postoperative cases [18]. This distinct microbiological profile of PTE emphasizes the need for pathogen-specific management strategies.

The primary management step in OGI is to close the wound as soon as possible, ideally within 24 h. Foreign bodies, especially those contaminated with soil, should be removed early [19]. Most cases of post-traumatic endophthalmitis are treated with immediate pars plana vitrectomy (PPV) [20,21]. Currently, there is no standardized protocol for the use of intravitreal antibiotics (IVAB). However, several studies have demonstrated that IVAB reduces the risk of endophthalmitis by up to eightfold compared to systemic prophylaxis alone while awaiting culture results [22,23]. Intravitreal injections of vancomycin (1 mg/0.1 mL) and ceftazidime (2.25 mg/0.1 mL), or amikacin (400 mg/0.1 mL), are recommended when vitreous haze, vasculitis, or peripheral inflammation is present, especially in cases involving soil-contaminated intraocular foreign bodies (IOFB) [24].

The management of pediatric OGI-related endophthalmitis differs from adult cases, yet research in this area remains sparse, and treatment is often based on adult-derived data. This gap underscores the need for pediatric-focused studies to optimize prevention and treatment strategies in children [25].

Diagnosing pediatric PTE presents unique challenges, primarily due to children’s limited ability to articulate symptoms and difficulties in cooperating during clinical exams, which can lead to misdiagnosis or delayed treatment [26]. Pediatric OGI-related endophthalmitis can result in severe, long-term visual impairment, negatively impacting children’s quality of life, educational opportunities, and socioeconomic prospects. Furthermore, it places a significant burden on families and healthcare systems [27,28].

Despite its severity, pediatric-specific studies remain scarce, primarily because of the relative rarity of the condition, ethical constraints in conducting clinical trials with children, and challenges in obtaining sufficient sample sizes [29]. These barriers underscore the importance of developing tailored guidelines and treatment protocols to improve clinical outcomes and minimize complications in children.

Notably, the incidence of retinal detachment in pediatric PTE cases reaches 38%, markedly higher than the 8.3% observed in adults, highlighting the more severe course of PTE in children and the need for heightened clinical vigilance [2,30]. Current evidence on risk factors for endophthalmitis in pediatric OGI is limited, and the role of prophylactic IVAB during primary repair is still debated. Identifying clinical risk factors, such as baseline visual acuity, microbial virulence, intraocular pressure, and retinal detachment, may assist in stratifying high-risk pediatric cases.

The primary aim of this study is to bridge this critical gap by describing the clinical characteristics and pathogenic microorganisms in pediatric PTE cases based on a ten-year review conducted at the Eye and ENT Hospital of Fudan University. Additionally, we seek to provide reference data on antibiotic susceptibility and treatment regimens to guide the future management of pediatric PTE patients. Finally, we aim to identify independent risk factors associated with visual prognosis to help improve treatment outcomes in pediatric PTE.

## 2. Results

### 2.1. Study Design and Clinical Characteristics of Pediatric PTE Patients

**Figure 1:**
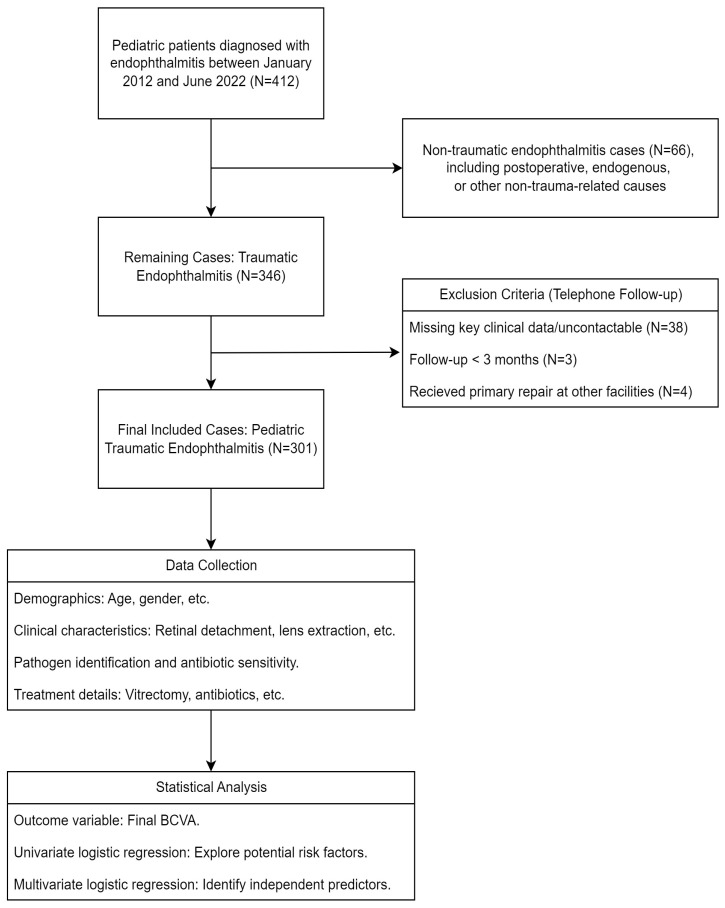
Study design flowchart for pediatric post-traumatic endophthalmitis (PTE) analysis. This flowchart (created using draw.io software, v24.2.5) outlines the stepwise selection process and study design for pediatric patients diagnosed with endophthalmitis between January 2012 and June 2022. The final cohort comprised 301 cases of pediatric PTE, with subsequent data collection and statistical analysis performed to evaluate clinical characteristics, pathogen profiles, treatment modalities, and visual outcomes.

The study design is illustrated in Figure 1, which outlines the stepwise process of patient selection, data collection, and analysis. This retrospective cohort study included pediatric patients diagnosed with post-traumatic endophthalmitis (PTE) between January 2012 and June 2022.

A total of 301 eyes from 301 pediatric patients diagnosed with post-traumatic endophthalmitis (PTE) were included in this study. Of these, 206 (68.4%) were male and 95 (31.6%) were female. The mean age of the cohort was 6.15 ± 3.40 years, with an age range of 0 to 18 years. Table 1 outlines the demographic and clinical profiles of the study population. Notably, 135 children (44.8%) sustained trauma involving metallic objects. Abnormal intraocular pressure was documented in 127 patients (42.2%), while traumatic cataracts were identified in 204 patients (67.8%). Additionally, 27 patients (9.0%) exhibited retinal detachment, 134 (44.5%) presented with corneal edema, and intraocular hemorrhage was observed in 38 patients (12.6%). The average length of hospital stay for these pediatric PTE cases was 5 days, ranging from 0 to 61 days.

### 2.2. Pathogenic Microorganisms and Antibiotic Sensitivity Results

Among the 301 pediatric post-traumatic endophthalmitis (PTE) cases, 142 cultures yielded positive results, representing a pathogen positivity rate of 47.2%. Vitreous samples tested positive in 89 cases, with a positivity rate of 29.6%, while aqueous humor samples tested positive in 34 cases, corresponding to a positivity rate of 11.3%. Gram-positive cocci were the predominant pathogens, accounting for 71.1% of positive cases, with *Viridans group streptococci* being the most common species, identified in 16 cases (11.3%). Gram-positive bacilli comprised 12.0% of the cases, with *Bacillus cereus* being the most frequently isolated, found in 13 cases (9.2%). Gram-negative bacilli accounted for 10.6% of the positive cultures, while fungi were identified in 6.3% of cases. Comprehensive findings are presented in Table 2.

Regarding antibiotic sensitivity, Gram-positive cocci exhibited high sensitivity to vancomycin (95.4%) and levofloxacin (84.3%), with moderate sensitivity to clindamycin (53.6%), and lower sensitivity to erythromycin (35.7%) and penicillin (50.0%). Gram-positive bacilli showed complete sensitivity to levofloxacin (100.0%) and good sensitivity to vancomycin (80.0%), while sensitivity to clindamycin (25.0%) was low, and sensitivity to erythromycin (71.4%) and penicillin (50.0%) were moderate. In contrast, Gram-negative bacilli were universally sensitive to meropenem, amikacin, and levofloxacin (all 100.0%), with slightly lower sensitivity to ceftazidime (83.3%). Table 3 provides a detailed overview of the results.

### 2.3. Treatment of Pediatric PTE Patients

Out of the 301 eyes from pediatric PTE patients, 216 eyes (71.8%) underwent vitrectomy. Emergency or immediate vitrectomy within 24 h of admission was performed on 171 eyes (56.8%), while 85 eyes (28.2%) did not receive vitrectomy. Overall, 247 vitrectomies were conducted, averaging 0.82 ± 0.99 per eye. Lens extraction was performed in 230 eyes (76.4%). The initial intravitreal antibiotic treatment, typically a combination of ceftazidime and norvancomycin, was administered in 248 cases (82.4%). A total of 393 intravitreal injections were given across the 301 cases, with an average of 1.30 ± 0.88 injections per eye.

Systemic antibiotics were administered in 265 cases (88.0%), with *ceftazidime* being the most commonly used (231 cases, 76.7%). The average duration of intravenous antibiotic administration was 6.02 ± 3.72 days. Systemic corticosteroids were used in 89 cases (29.6%), with dexamethasone being the most frequently administered corticosteroid (81 cases, 91.0%). The average duration of intravenous dexamethasone administration was 5.02 ± 2.16 days. Further details are provided in Table 4.

### 2.4. Univariate Analysis of Factors Affecting Post-Treatment Visual Outcomes in PTE Patients

The median follow-up period was 26 months, ranging from 3 months to 8 years. Based on the best-corrected visual acuity (BCVA) at the final follow-up, patients were categorized into two groups: the vision recovery group (BCVA ≥ 0.05) and the vision non-recovery group (BCVA < 0.05). Prior to treatment, 43 patients (14.3%) had a BCVA of ≥0.05, whereas after treatment, 161 patients (53.5%) achieved a final BCVA of ≥0.05. A total of 87 patients (28.9%) had a final BCVA of <0.05, including 9 eyes (3.0%) that eventually required evisceration. For 53 patients (17.6%), final follow-up BCVA data were unavailable. Comprehensive data are provided in the Supplementary Material.

**Figure 2:**
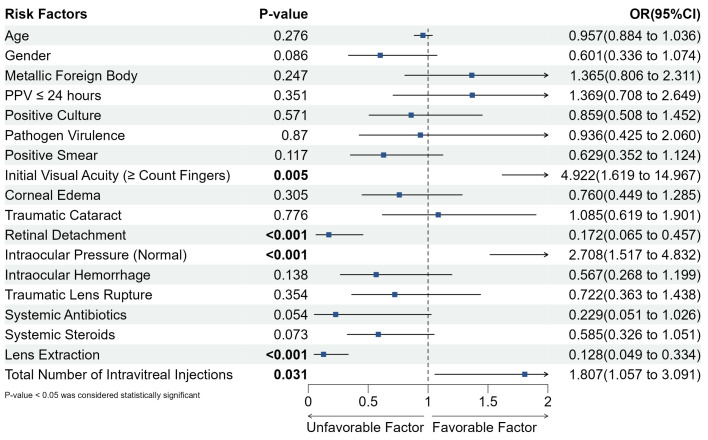
Risk factors for visual prognosis in pediatric endophthalmitis. This forest plot displays the odds ratios (ORs) and 95% confidence intervals (CIs) for each risk factor associated with visual prognosis in pediatric endophthalmitis. Significant associations (*p* < 0.05) are highlighted in bold, indicating factors with a statistically significant impact on the outcome. Key findings include a significant association of initial visual acuity (≥Count Fingers), retinal detachment, normal intraocular pressure, lens extraction, and total number of intravitreal injections with the visual prognosis. OR values above 1 suggest a favorable factor, while values below 1 indicate an unfavorable factor.

Univariate analysis was conducted to assess various risk factors influencing visual prognosis in pediatric post-traumatic endophthalmitis (PTE) patients. Factors included age, gender, trauma from metallic objects, timing of pars plana vitrectomy (PPV) within 24 h, positive culture results, pathogen virulence, pathogen detection in smears, initial visual acuity (≥counting fingers), presence of corneal edema, traumatic cataracts, retinal detachment, normal intraocular pressure, intraocular hemorrhage, traumatic lens rupture, use of systemic antibiotics and steroids, timing of trauma repair, lens extraction, and the number of intravitreal antibiotic injections. Statistically significant factors associated with improved best-corrected visual acuity (BCVA) (*p* < 0.05) were initial visual acuity (≥counting fingers), the absence of retinal detachment, normal intraocular pressure, lens extraction, and the total number of intravitreal injections. Full statistical outcomes are provided in Figure 2.

Further, factors with a *p*-value of <0.05 from the univariate regression analysis were included in the multivariate regression model to identify the independent predictors of visual prognosis in pediatric endophthalmitis. Prior to finalizing the multivariate model, we assessed multicollinearity using the “condition number” method from the “kappa function” in R. The condition number (condition number = 22.637) indicated a high level of multicollinearity among certain variables, with initial visual acuity showing particularly strong collinearity with other variables. As a result, initial visual acuity was excluded from the multivariate model to improve model stability and interpretability.

**Figure 3:**
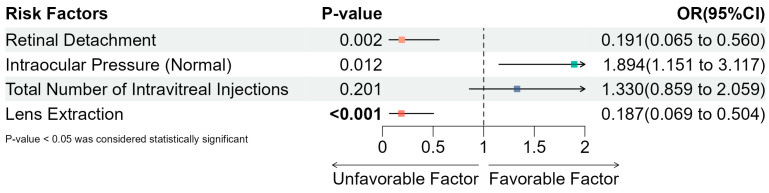
Independent risk factors influencing visual prognosis in pediatric endophthalmitis patients. This forest plot presents the odds ratios (ORs) with 95% confidence intervals (CIs) for each variable in the multivariate regression model, assessing the independent impact of various risk factors on visual prognosis. Significant factors (*p* < 0.05) include retinal detachment (OR = 0.191, 95% CI: 0.065–0.560, *p* = 0.002), normal intraocular pressure (OR = 1.894, 95% CI: 1.151–3.117, *p* = 0.012), and lens extraction (OR = 0.187, 95% CI: 0.069–0.504, *p* < 0.001), indicating that these factors are independently associated with the outcome. The dashed vertical line at OR = 1 represents the no-effect threshold, with values to the left indicating a potential negative effect on prognosis, while values to the right suggest a favorable effect. Non-significant factors (*p* ≥ 0.05) are also shown for comparison.

The final multivariate regression analysis results are presented in Figure 3. Significant independent predictors included retinal detachment (OR = 0.191, 95% CI: 0.065–0.560, *p* = 0.002), normal intraocular pressure (OR = 1.894, 95% CI: 1.151–3.117, *p* = 0.012), and lens extraction (OR = 0.187, 95% CI: 0.069–0.504, *p* < 0.001). Retinal detachment and lens extraction were associated with a lower likelihood of a favorable visual outcome, whereas normal intraocular pressure was positively associated with improved prognosis. These findings underscore the importance of managing retinal detachment and intraocular pressure to potentially improve visual outcomes in pediatric endophthalmitis, while avoiding unnecessary lens extraction when feasible.

## 3. Discussion

This study analyzed 301 cases of pediatric post-traumatic endophthalmitis (PTE) diagnosed over a ten-year period at the Eye and ENT Hospital of Fudan University, revealing that despite aggressive anti-infective therapies, only 161 patients (53.49%) achieved a final best-corrected visual acuity (BCVA) of ≥0.05. These findings emphasize the severe visual outcomes commonly associated with pediatric PTE and underscore the complexity of its management, highlighting the critical need for targeted interventions to improve recovery prospects in this vulnerable population.

Microbiological culture remains the “gold standard” for diagnosing infectious endophthalmitis [31]. Ocular trauma is a leading cause of infectious endophthalmitis in developing countries, responsible for over 58.0–80.0% of cases [32]. Previous studies have reported culture positivity rates for clinically diagnosed infectious endophthalmitis ranging from 31.8% to 63.0% [6,33,34]. In this cohort, 142 cases (47.2%) were culture-positive, aligning with the existing literature. Gram-positive cocci were the predominant pathogens (71.1%), with *Viridans group streptococci* identified as the most common organism (16 cases, 11.3%). Gram-positive bacilli accounted for 12.0%, Gram-negative bacilli for 10.7%, and fungi for 6.3% of the positive cultures, consistent with previous reports [35].

Our study demonstrated that fluoroquinolones exhibit high sensitivity against both Gram-positive bacilli and Gram-negative bacteria, consistent with findings from previous research. For instance, Yang et al. observed that fluoroquinolones were highly effective against a range of pathogens commonly implicated in pediatric endophthalmitis, highlighting their broad-spectrum antibacterial efficacy [36]. However, the clinical application of fluoroquinolones in pediatric patients warrants careful consideration due to potential adverse effects unique to this age group. Notably, previous studies have documented risks such as cartilage damage and musculoskeletal issues, particularly arthropathy, associated with fluoroquinolone use in children [37]. These potential side effects underscore the need for a balanced approach in prescribing fluoroquinolones, especially in pediatric cases where long-term skeletal health is a priority.

As previously noted, there is currently a lack of standardized treatment guidelines specifically for pediatric cases of post-traumatic endophthalmitis (PTE). The treatment protocol applied in this study was based on our institution’s established approach, which aligns with the ’Chinese Expert Consensus on the Diagnosis and Treatment of Postoperative Infectious Endophthalmitis’ (2022). This protocol was rigorously implemented in each case, providing a structured approach in the absence of pediatric-specific guidelines.

Our analysis demonstrated that the absence of retinal detachment (OR = 0.191, 95% CI: 0.065–0.560, *p* = 0.002), the maintenance of normal intraocular pressure (OR = 1.894, 95% CI: 1.151–3.117, *p* = 0.012), and the absence of lens extraction (OR = 0.187, 95% CI: 0.069–0.504, *p* < 0.001) were independently associated with favorable visual outcomes. These findings align with recent studies, such as those by Nicoară et al. and Hapca et al., which identified retinal detachment and delayed treatment as significant negative prognostic factors in traumatic endophthalmitis cases [38,39]. Excluding initial visual acuity from our analysis underscores the complexity of pediatric endophthalmitis prognosis, where structural and procedural factors, such as retinal attachment and intraocular pressure, may play more decisive roles in outcomes than initial visual acuity alone.

Additionally, Watanachai et al. found that intraocular foreign bodies and retinal injury significantly correlated with poor outcomes in PTE, emphasizing the importance of retinal integrity in these cases [3]. This suggests that early, targeted surgical interventions and vigilant intraocular pressure management are crucial for optimizing outcomes in pediatric PTE.

The retrospective nature of this study introduces certain limitations, including potential biases in data collection and variability in follow-up durations and treatment regimens. Additionally, missing data, particularly the absence of follow-up visual acuity for 17.61% of patients, may have influenced the assessment of treatment outcomes and introduced reporting bias. This limitation underscores the need for prospective studies with more standardized and comprehensive data collection protocols to reduce variability and improve accuracy.

Furthermore, resource constraints prevented the routine use of polymerase chain reaction (PCR) testing for pathogen detection, which could have enhanced diagnostic precision, particularly in culture-negative cases. Introducing PCR technology into clinical diagnostics holds great promise, as it enables the rapid and sensitive identification of pathogens, even in cases with low microbial loads or atypical organisms, thereby improving the accuracy of early diagnoses and guiding more targeted treatment strategies.

In conclusion, Gram-positive cocci were identified as the primary pathogens in pediatric PTE. Early pars plana vitrectomy (PPV), particularly within 24 h of hospitalization, combined with targeted intravitreal antibiotics such as ceftazidime and vancomycin, remains the most effective treatment strategy for managing pediatric PTE cases. Our findings demonstrate that favorable visual outcomes are strongly associated with the absence of retinal detachment, normal intraocular pressure, and the avoidance of lens extraction.

To improve pediatric PTE management, clinicians should prioritize early surgical intervention, vigilant intraocular pressure control, and the timely administration of antibiotics. These findings highlight the need for standardized pediatric-specific treatment protocols to optimize outcomes.

Future research should focus on conducting prospective studies to validate these findings and address the limitations of retrospective analyses. Additionally, there is an urgent need to develop pediatric-specific treatment protocols tailored to the unique challenges of managing PTE in children, such as early diagnosis, timely intervention, and long-term follow-up.

## 4. Materials and Methods

### 4.1. Study Population

We conducted a retrospective, single-center case series study involving 301 eyes from 301 children (aged ≤ 18 years) diagnosed with endophthalmitis at the Eye and ENT Hospital of Fudan University, Shanghai, China, between January 2012 and June 2022. Patients were initially identified through medical records based on a clinical diagnosis of endophthalmitis, and further screening was performed to confirm whether the cases were post-traumatic in origin. Exclusion criteria included substantial missing data, an inability to establish follow-up via telephone, follow-up durations of less than three months, and cases that had received primary treatment at other institutions. Ethical approval for this study was obtained from the Institutional Review Board (Approval No. 2022170), and all procedures adhered to the principles of the Declaration of Helsinki. To ensure confidentiality and privacy, patient data were anonymized in compliance with ethical guidelines.

### 4.2. Study Design and Methods

#### 4.2.1. Data Collection

Data were collected from electronic medical records, including patient history, clinical characteristics, causative microorganisms, treatment modalities, and best-corrected visual acuity (BCVA).

#### 4.2.2. Data Analysis

Collected data were organized and categorized using Excel, then subjected to statistical analysis. For cases with missing information, follow-up was conducted via telephone to obtain the necessary data.

### 4.3. Treatment of Pediatric Endophthalmitis

#### 4.3.1. Pars Plana Vitrectomy

For disinfection, 5% povidone-iodine was applied to the conjunctival sac. All vitrectomies were performed using 23 G or 25 G pars plana vitrectomy (PPV), with conjunctival incisions that did not require sutures. PPV was considered if there was significant clinical deterioration within 24 h of the initial diagnosis or if the condition did not improve within 48 h after the first intravitreal antibiotic injection. In cases involving an intraocular foreign body (IOFB), traumatic retinal detachment, or lens displacement, early vitrectomy was performed. Additional surgical procedures could be combined with PPV as needed, depending on the clinical presentation. General anesthesia was administered for all vitrectomy procedures to ensure patient comfort and immobility during surgery, given the age of the pediatric population.

#### 4.3.2. Intravitreal Injection

Most patients received intravitreal antibiotic injections immediately following diagnosis. The antibiotics commonly used included norvancomycin (1 mg/0.1 mL) in combination with ceftazidime (2.25 mg/0.1 mL), with or without corticosteroids such as dexamethasone (0.4 mg/0.1 mL), triamcinolone acetonide (1 mg/0.1 mL), or methylprednisolone sodium succinate (40 mg/0.1 mL). Intensive treatment also involved the use of topical eye drops, including 0.3% ofloxacin three times daily, 0.3% tobramycin three times daily, 0.3% gatifloxacin three times daily, and 1% prednisolone acetate or 0.1% fluorometholone three times daily. Intravenous ceftazidime (1.5 g twice daily for 7–10 days) was also administered. Local anesthesia (topical or subconjunctival) was used for intravitreal injections to minimize discomfort while ensuring the precision of the procedure, particularly in cases where general anesthesia was not required.

For fungal infections confirmed by microscopy or culture from vitreous or other samples, intravitreal amphotericin B (5 μg/0.1 mL) or voriconazole (100 μg/0.1 mL) was administered, alongside systemic voriconazole (200 mg once daily for 15 days) or itraconazole (200 mg twice daily for 15 days). Topical eye drops (0.2% fluconazole three times daily, 0.5% voriconazole every two hours, or 0.5% amphotericin B four times daily) were applied according to the patient’s condition. If there was no significant improvement after these treatments, repeated vitrectomy or intravitreal antibiotic injections were considered.

In suspected fungal endophthalmitis cases, empirical intravitreal antifungal injections were administered immediately after vitreous or aqueous samples were collected, even prior to a definitive diagnosis. If a vitreous biopsy was initially performed, further diagnostic vitrectomy might be required. Although intravitreal antibiotics were administered during initial treatment, excised vitreous samples could still test positive for fungal growth.

#### 4.3.3. Microbiological Sample Collection and Culture Protocol

Undiluted vitreous and aqueous samples were collected via needle aspiration or vitrectomy before surgery or injection in all cases. Samples were sent to the microbiology laboratory at Eye and ENT Hospital of Fudan University for direct smear and culture. For cases requiring corneal transplantation due to fungal keratitis, aqueous samples were collected intraoperatively. In cases where corneal involvement impaired vision, vitreous aspiration was performed instead of vitrectomy. Vitreous samples (1–1.5 mL) were collected using a vitreous cutter at 5000 cuts per minute with a 2 mL syringe under manual suction. Smears were stained using 0.1% calcofluor white, Gram stain, and Gomori’s methenamine silver (GMS) stain. Cultures were performed on 5% sheep blood agar, chocolate agar, thioglycollate broth, brain heart infusion broth, Sabouraud dextrose agar, and potato dextrose agar. Sabouraud dextrose and potato dextrose agars were incubated at 27 °C for two weeks, while chocolate agar was incubated at 37 °C in a 5% CO_2_ environment. Other media were incubated aerobically at 37 °C.

#### 4.3.4. Antibiotic Sensitivity Testing

Antibiotic sensitivity testing was performed using the modified Kirby–Bauer (K-B) disk diffusion method, following the guidelines of the National Clinical Laboratory Standards (NCCLS). Antibiotic disks were supplied by OXOID Ltd., Basingstoke, UK. Bacteria were spread on Mueller–Hinton (MH) agar plates and incubated at 35–37 °C for 24 h. Antibiotic sensitivity was determined based on the diameter of the inhibition zones, according to NCCLS.

### 4.4. Statistical Methods

All data were entered into Microsoft Excel and analyzed using SPSS software (version 22.0; IBM Corp., Armonk, NY, USA) and R software (version 4.3.2; R Foundation for Statistical Computing, Vienna, Austria). Continuous variables were presented as mean ± standard deviation (SD). The impact of continuous variables on final visual acuity was evaluated using the *t*-test, while categorical variables were compared using the chi-square test. Univariate and multivariate regression analyses were conducted in R to examine the associations between independent variables and outcomes. Logistic regression was employed to assess the relationship between continuous independent variables and categorical dependent variables. A *p*-value of <0.05 was considered statistically significant.

### 4.5. Manuscript Preparation

The preparation of this manuscript involved the use of ChatGPT for drafting and language editing purposes. All AI-assisted content was thoroughly reviewed and revised by the authors to ensure the originality and integrity of the work.

## Figures and Tables

**Table 1:** Demographic and clinical characteristics of the patients.

Characteristics	Total (*n* = 301)	1–3 Years (*n* = 73)	4–12 Years (*n* = 213)	13–18 Years (*n* = 15)
Age, mean ± SD	6.15 ± 3.40	2.26 ± 0.85	6.86 ± 2.18	14.93 ± 1.67
Gender				
Male	206 (68.4%)	45 (61.6%)	147 (69.0%)	14 (93.3%)
Female	95 (31.6%)	28 (38.4%)	66 (31.0%)	1 (6.7%)
Affected Eye				
Left Eye	131 (43.5%)	33 (45.2%)	93 (43.7%)	5 (33.3%)
Right Eye	170 (56.5%)	40 (54.8%)	120 (56.3%)	10 (66.7%)
Metallic Object Injury	135 (44.8%)	37 (50.7%)	90 (42.2%)	8 (53.3%)
Intraocular Pressure				
>20 mm Hg	19 (6.3%)	2 (2.7%)	16 (7.5%)	1 (6.7%)
10–20 mm Hg	139 (46.2%)	44 (60.3%)	89 (41.8%)	6 (40.0%)
<10 mm Hg	108 (35.9%)	17 (23.3%)	83 (39.0%)	8 (53.3%)
Missing Data	35 (11.0%)	10 (13.7%)	25 (11.7%)	
Admission Visual Acuity				
≥FC ^1^	43 (14.3%)			
<FC ^2^	120 (39.9%)			
Non-Cooperative (Unassessable)	138 (45.8%)			
Corneal Edema	134 (44.5%)	33 (45.2%)	95 (44.6%)	6 (40.0%)
Traumatic Cataract	204 (67.8%)	44 (60.3%)	148 (69.5%)	12 (80.0%)
Retinal Detachment	27 (9.0%)	8 (11.0%)	16 (7.51%)	3 (20.0%)
Intraocular Hemorrhage	38 (12.6%)	5 (6.8%)	31 (14.6%)	2 (13.3%)
Source of Positive Microbial Smear	75 (24.9%)	18 (24.6%)	54 (25.4%)	3 (20.0%)
Vitreous	36 (12.0%)	7 (9.6%)	26 (12.2%)	3 (20.0%)
Aqueous	11 (3.6%)	5 (6.8%)	6 (2.8%)	
Others	28 (9.3%)	6 (8.2%)	22 (10.3%)	
Source of Positive Microbial Culture	133 (44.2%)	23 (31.5%)	97 (45.5%)	13 (86.7%)
Vitreous	65 (21.6%)	11 (15.1%)	49 (23.0%)	5 (33.3%)
Aqueous	25 (8.3%)	8 (11.0%)	15 (7.0%)	2 (13.3%)
Intraocular Foreign Body	13 (4.3%)	2 (2.7%)	7 (3.3%)	4 (26.7%)
Eye Discharge	3 (1.0%)		3 (1.4%)	
Pus	4 (1.3%)	2 (2.7%)	2 (0.9%)	
Others	23 (7.6%)		21 (9.8%)	2 (13.3%)
Hospital Stay (days, mean ± SD)	4.86 ± 4.61	4.39 ± 2.92	5.14 ± 5.15	3.08 ± 2.12

Data are *n* (%) unless otherwise specified. ^1^ ≥FC: Best-corrected visual acuity (BCVA) equal to or better than Finger Counting on admission. ^2^ <FC: Best-corrected visual acuity (BCVA) worse than Finger Counting on admission.

**Table 2:** Age distribution of pathogenic organisms in pediatric PTE patients.

Pathogenic Organisms	Total (*n* = 142)	0–3 Years (*n* = 30)	4–12 Years (*n* = 104)	13–17 Years (*n* = 8)
Gram-Positive Cocci	101 (71.1%)	24 (80.0%)	72 (69.2%)	5 (62.5%)
*Viridans group streptococci*	16 (11.3%)	2 (6.7%)	14 (13.5%)	
*Streptococcus pneumoniae*	6 (4.2%)	4 (13.3%)	2 (1.9%)	
*Group B Streptococcus*	4 (2.8%)	2 (6.7%)	2 (1.9%)	
*D Group Streptococcus*	2 (1.4%)	1 (3.3%)	1 (1.0%)	
*Streptococcus constellatus*	1 (0.7%)		1 (1.0%)	
*Staphylococcus capitis*	2 (1.4%)		2 (1.9%)	
*Staphylococcus xylosus*	1 (0.7%)		1 (1.0%)	
*Staphylococcus epidermidis*	14 (9.9%)	4 (13.3%)	8 (7.7%)	2 (25.0%)
*Staphylococcus warneri*	6 (4.2%)	2 (6.7%)	4 (3.8%)	
*Staphylococcus hominis*	1 (0.7%)		1 (1.0%)	
*Staphylococcus auricularis*	1 (0.7%)		1 (1.0%)	
*Staphylococcus aureus*	7 (4.9%)		6 (5.8%)	1 (12.5%)
*Staphylococcus lentus*	2 (1.4%)		2 (1.9%)	
*Staphylococcus haemolyticus*	2 (1.4%)		2 (1.9%)	
*Staphylococcus cohnii*	1 (0.7%)	1 (3.3%)		
*Enterococcus faecalis*	1 (0.7%)	1 (3.3%)		
*Other Gram-Positive Cocci*	34 (23.9%)	7 (23.3%)	25 (24.0%)	2 (25.0%)
Gram-Positive Bacilli	17 (12.0%)	3 (10.0%)	12 (11.5%)	2 (25.0%)
*Bacillus cereus*	13 (9.2%)	3 (10.0%)	8 (7.7%)	2 (25.0%)
*Bacillus subtilis*	3 (2.1%)		3 (2.9%)	
*Other Gram-Positive Bacilli*	1 (0.7%)		1 (1.0%)	
Gram-Negative Bacteria	15 (10.6%)	1 (3.3%)	14 (13.5%)	
*Acinetobacter lwoffii*	4 (2.8%)		4 (3.8%)	
*Proteus vulgaris*	1 (0.7%)		1 (1.0%)	
*Serratia marcescens*	1 (0.7%)		1 (1.0%)	
*Enterobacter cloacae*	3 (2.1%)		3 (2.9%)	
*Enterobacter agglomerans*	2 (1.4%)		2 (1.9%)	
*Klebsiella pneumoniae*	1 (0.7%)		1 (1.0%)	
*Vibrio* spp.	1 (0.7%)		1 (1.0%)	
*Escherichia hermannii*	1 (0.7%)		1 (1.0%)	
*Other Gram-Negative Bacteria*	1 (0.7%)	1 (3.3%)		
Fungi	9 (6.3%)	2 (6.7%)	6 (5.8%)	1 (12.5%)
*Aspergillus fumigatus*	1 (0.7%)			1 (12.5%)
*Other Aspergillus species*	3 (2.1%)	1 (3.3%)	2 (1.9%)	
*Rhodotorula mucilaginosa*	1 (0.7%)		1 (1.0%)	
*Fusarium* spp.	1 (0.7%)		1 (1.0%)	
*Other Fungi*	3 (2.1%)	1 (3.3%)	2 (1.9%)	

Data are *n* (%) unless otherwise specified.

**Table 3:** Antibiotic sensitivity of isolated pathogens in pediatric PTE patients.

Antibiotics	Gram-Positive Cocci (*n* = 70)	Gram-Positive Bacilli (*n* = 15)	Gram-Negative Bacteria (*n* = 14)
**Penicillin**	35/70	4/15	
Amoxicillin	17/30		
Ampicillin	42/70		1/9
Cefuroxime	10/19		3/9
Ceftriaxone	18/32		8/10
Cefoxitin	9/13	0/10	6/8
Cefepime	9/17		9/12
Cefotaxime			9/11
**Ceftazidime**			10/12
Erythromycin	25/70	10/14	
Chloramphenicol	34/40	9/11	
Gentamicin	26/32		11/12
Tobramycin		7/9	10/10
**Amikacin**			12/12
Ciprofloxacin	45/65	12/14	11/12
Moxifloxacin	27/30		
**Levofloxacin**	59/70	11/11	12/12
Ofloxacin	36/40	11/12	
Tetracycline	30/45	8/10	
**Ertapenem**			8/8
**Meropenem**			12/12
Imipenem			7/9
**Clindamycin**	30/56	2/8	
**Vancomycin**	63/66	12/15	
Daptomycin	30/30		
Rifampicin	44/47	1/10	
TMP-SMZ			10/12

Data are presented as *n*/N, indicating the number of sensitive isolates out of the total tested for each antibiotic. Antibiotic sensitivity was assessed using the modified Kirby–Bauer disk diffusion method, following NCCLS/CLSI standards. Commonly used antibiotics and those with high sensitivity (≥90% of isolates sensitive) are highlighted in bold for emphasis.

**Table 4:** Medical interventions and antibiotic use in pediatric endophthalmitis patients.

Treatment Method	Total (*n* = 301)	0–3 Years (*n* = 73)	4–12 Years (*n* = 213)	13–18 Years (*n* = 15)
Timing of PPV				
PPV > 24 h ^1^	45 (15.0%)	15 (20.6%)	30 (14.1%)	
PPV ≤ 24 h ^2^	171 (56.8%)	33 (45.2%)	128 (60.1%)	10 (66.7%)
No PPV	85 (28.2%)	25 (34.2%)	55 (25.8%)	5 (33.3%)
Total Vitrectomies, sessions	247	53	184	10
Vitrectomy sessions, mean ± SD	0.82 ± 0.99	0.73 ± 0.45	0.86 ± 1.14	0.67 ± 0.49
Lens Extraction	230 (76.4%)	57 (78.1%)	158 (74.2%)	15 (100.0%)
Initial Intravitreal Injection of Antibiotics				
Ceftazidime + Vancomycin	3 (1.0%)		3 (1.4%)	
Ceftazidime + Norvancomycin ^3^	248 (82.4%)	57 (78.1%)	178 (83.6%)	13 (86.7%)
Ceftazidime + Norvancomycinn + Other	14 (4.6%)	6 (8.2%)	7 (3.3%)	1 (6.7%)
Norvancomycin + Other	7 (2.3%)	1 (1.4%)	6 (2.8%)	
Norvancomycin	26 (8.6%)	8 (11.0%)	17 (8.0%)	1 (6.7%)
Amphotericin B	3 (1.0%)	1 (1.4%)	2 (0.9%)	
Total Number of Intravitreal Injections, sessions	393	95	281	17
Intravitreal Antibiotic Injections, mean ± SD	1.30 ± 0.88	1.30 ± 0.46	1.32 ± 1.00	1.13 ± 0.35
Systemic Antibiotics Regimen				
Ceftazidime	231 (76.7%)	57 (78.1%)	164 (77.0%)	10 (66.7%)
Ceftriaxone	22 (7.3%)	4 (5.5%)	16 (7.5%)	2 (13.3%)
Other antibiotics	12 (4.0%)	4 (5.5%)	7 (3.3%)	1 (6.7%)
No systemic antibiotics	36 (12.0%)	8 (11.0%)	26 (12.2%)	2 (13.3%)
Topical Antibiotics	301 (100.0%)	73 (100.0%)	213 (100.0%)	15 (100.0%)
Intravitreal Steroid Injections	52 (17.3%)	9 (12.3%)	37 (17.4%)	6 (40.0%)
Systemic Steroids	89 (29.6%)	25 (34.2%)	59 (27.7%)	5 (33.3%)
Topical Steroids	223 (74.1%)	53 (72.6%)	164 (77.0%)	6 (40.0%)

Data are *n* (%) unless otherwise specified. ^1^ PPV > 24 h: PPV performed more than 24 h after admission. ^2^ PPV ≤ 24 h: PPV performed within 24 h after admission. ^3^ Norvancomycin: A glycopeptide antibiotic similar to vancomycin, used as an alternative for treating Gram-positive bacterial infections.

## Data Availability

The data presented in this study are available on request from the corresponding author. The data are not publicly available due to privacy or ethical restrictions.

## Supplementary Materials

The following supporting information can be downloaded at: https://www.mdpi.com/article/10.3390/antibiotics14010020/s1. A supplementary table is provided, detailing patient demographic and clinical characteristics, including variables such as age, sex, ocular conditions, treatments, microbiological findings, and visual outcomes. This table supports the interpretation of the study’s findings and offers additional insights into the dataset analyzed.
